# Identification of patients at risk for adverse events and poor symptom improvement after transcatheter aortic valve implantation

**DOI:** 10.1016/j.ahjo.2026.100735

**Published:** 2026-02-02

**Authors:** Kees van Bergeijk, Constantijn (Stijn) Venema, Bob Ophuis, Luca Plekkenpol, Mara Tomei, Hayman Al-Barwary, Jasper Tromp, Yoran Hummel, Wouter Ouwerkerk, Ad van den Heuvel, Hindrik van der Werf, Yvonne Douglas, Dajiro Tomii, Thomas Pilgrim, Stephan Windecker, Adriaan Voors, Joanna Wykrzykowska

**Affiliations:** a Department of Cardiology, University of Groningen, University Medical Centre Groningen, Groningen, the Netherlands; b Saw Swee Hock School of Public Health, National University of Singapore, and the National University Health System, Singapore, Singapore; c Department of Dermatology, University of Amsterdam Medical Centre, Amsterdam, Netherlands; d Department of Cardiology, Inselspital, University of Bern, Bern, Switzerland; e Us2.ai, Singapore, Singapore

**Keywords:** TAVI, Symptoms, Adverse outcomes, Echocardiography, AI derived parameters

## Abstract

**Background:**

Transcatheter aortic valve implantation (TAVI) aims to improve symptoms and prognosis, while minimising adverse outcomes. Available prediction models focus on individual outcomes, but those combining adverse events and symptom improvement in a single prediction model are scarce, and include only few variables and lack external validation. Using machine learning, we developed a clinically relevant model to identify patients at high risk of both adverse events and poor symptom improvement after TAVI.

**Methods:**

In total, 72 candidate variables including clinical, medication use, biomarkers and (AI-derived) echocardiographic parameters were collected in patients with severe symptomatic AS undergoing TAVI. The primary outcome was a combination of poor symptom improvement (NYHA compared with baseline) and a composite of cardiovascular mortality, stroke or heart failure hospitalisation) at one year follow-up. LASSO Logistic regression was used for variable selection. External validation was performed in the Bern TAVI-registry.

**Results:**

From a total of 827 patients (age 79.2 (± 7.29), 53% female), 101 patients (12%) had both adverse events and poor symptom improvement during one-year follow-up after TAVI, while 529 (64%) improved without any adverse events. Predictors for the combined primary outcome were history of COPD, use of vitamin-K antagonist, concomitant heart failure, reflected by mineralocorticoid receptor antagonists use, lower sodium and higher urea and (log-)NT-proBNP levels, lower AV mean gradient and larger LVOT diameter (area under the curve (AUC): 0.74 (internal validation: 0.72 and external validation: 0.66)).

**Conclusion:**

Our externally validated model can reasonably identify patients with both poor symptom improvement and adverse events after TAVI.

## Graphical abstract

Predictors for poor symptom improvement and adverse events one year post-TAVI. Main findings of this retrospective observational cohort study, showing predictors for no symptom improvement and adverse events one-year post-TAVI. AV P Mean: Aortic Valve Mean Gradient, COPD: Chronic Obstructive Pulmonary Disease, MRA: Mineralocorticoid Receptor Antagonist, LVOT: Left ventricular outflow tract, NT-proBNP: N-terminal pro-B-type natriuretic peptide, OR: Odds Ratio, Vit. K ant: vitamin K antagonist.

**Figure:**
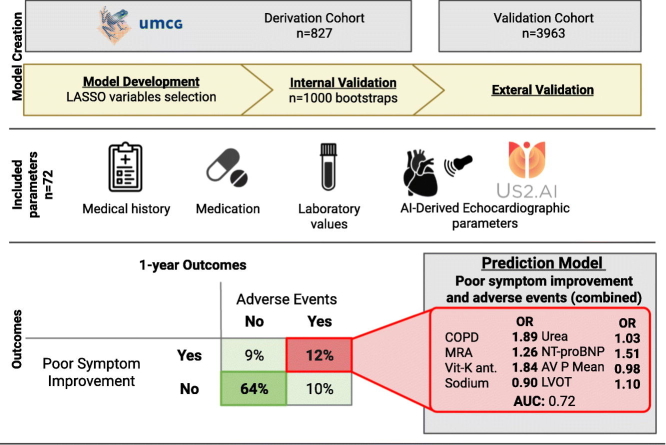

## Abbreviations and acronyms

**Table:** 

AV Mean Gradient	Aortic Valve Mean Gradient
COPD	Chronic Obstructive Pulmonary Disease
MRA	Mineralocorticoid Receptor Antagonist
NT-proBNP	N-terminal pro-B-type natriuretic peptide
NYHA	New York Heart Association
TAVI	Transcatheter aortic valve implantation

## 1 Introduction

Severe aortic stenosis (AS) is often accompanied by dyspnea and is associated with increased mortality if left untreated [1]. Transcatheter aortic valve implantation (TAVI) has become the preferred treatment for AS in elderly or high-risk patients, and has recently demonstrated its feasibility and efficacy up to 5 years in low-risk patients as well [2], [3], [4], [5].

In this predominantly elderly TAVI population, symptom response may be the most important outcome from the patient's perspective. While most studies focus on hard endpoints after TAVI, such as mortality or stroke, only few studies have combined adverse events and symptom improvement as endpoints [6], [7]. For example, Lopes et al. investigated mortality and NYHA improvement separately at one year, whereas only Zusman and colleagues predicted a combined endpoint of mortality, stroke, and NYHA class improvement [8], [9]. These studies have limitations that make their result difficult to translate into clinical practice. This may be partly attributable to the fact that they only include a few pre-procedure candidate variables, lack external validation and do not take full advantage of novel machine learning modelling techniques [10]. AI-derived echocardiographic parameters may additionally increase power to predict outcomes in TAVI patients, as they are accurate and minimise inter-observer variability [11], [12]. Finally, most studies do not include hospitalisation for heart failure as an outcome, although this is still a common event during follow-up after TAVI, with reduced quality of life and increased mortality [13].

Therefore, the current study aimed to determine baseline predictors of the combined outcome of poor symptom improvement and adverse outcomes in a contemporary real-world TAVI cohort using clinical characteristics, biomarkers, and AI-derived echocardiographic parameters, with an external validation cohort. In this manner, we aimed to develop a personalised risk prediction model for combined outcomes after TAVI that could inform clinical decision making and patient shared decision making before the procedure and thus potentially avoid futile procedures.

## 2 Methods

### 2.1 Study design

The derivation cohort incorporated all patients who received a TAVI procedure in a tertiary centre (the University Medical Centre Groningen (UMCG)) between 27 May 2009 and 02 September 2020. Patients with an indication for TAVI other than severe native aortic stenosis were excluded from this analysis. All TAVI access types were eligible for inclusion, including transfemoral, subclavian, transapical and direct-aortic access. The approval for this study was obtained from the local research ethical committee and requirement for informed consent was waived (local number: 202100641).

External validation was performed in the Bern TAVI registry, a prospective registry cohort that included aortic stenosis patients treated with TAVI at the Bern University Hospital, Switzerland between 2009 and 2023 [14].

### 2.2 Data collection and management

Baseline clinical characteristics were retrospectively collected at the treating centre. The baseline visit was conducted (within) 1 month before the initial TAVI procedure and performed by a medical professional. Baseline characteristics were defined according to most recent guidelines (**Supplementary methods)**. Follow-up visits took place according to the local protocol at the treating centre after 30 days and at the referring hospitals 6 months and 12 months after TAVI procedure. Follow-up data was collected from electronic patient records at both treating and referring hospitals.

### 2.3 Outcomes

**Fig. 1:**
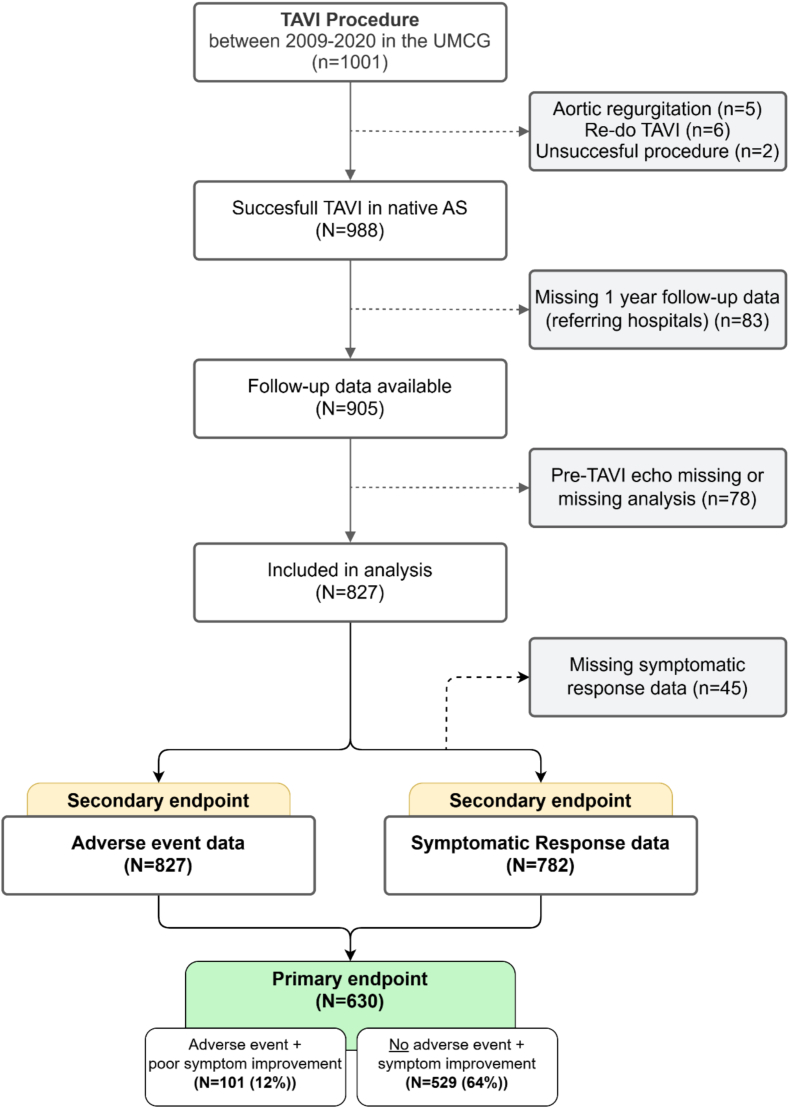
Study Flowchart.

The primary outcome was a combination of an adverse event and poor symptom improvement. Adverse events were defined according to the VARC-3 criteria as a composite of cardiovascular mortality, stroke, or heart failure hospitalisation within one year follow-up [15]. All cases of mortality in the derivation cohort were adjudicated by JJW and AAV. Poor symptom improvement was defined as the same or higher New York Heart Association (NYHA) class at 12 months compared to baseline [16]. Patients without available NYHA class at 12 months who died because of cardiovascular death within 30 days or valve or heart failure related death (with symptoms) within 365 days after the procedure were classified as poor symptom improvement. In the case of absence of 12 months (symptomatic) follow-up information, either 6 months or 30-day symptomatic information was taken **(S.** Fig. 1**)**. The secondary outcomes were: 1) adverse events and 2) poor symptom improvement separately.

### 2.4 Echocardiography and deep-learning based analysis

Transthoracic echocardiography was performed according to local guidelines before the TAVI procedure. Echocardiographic images were collected in Digital Imaging and Communications in Medicine (DICOM) format. To minimise inter-observer variability, the images were analysed with a fully automated, validated and clinical available deep learning-based echocardiography software (Us2.ai, version 2.0.0). This software has shown effective performance previously [11], [17], [18]. With the software, raw echocardiographic DICOM images are analysed using a convolutional neural network that correctly identify 2D or Doppler view and segment and annotate cardiac structures [19]. The software reports all conventional echocardiographic parameters (**S.** Table 1**)**. Before implementing the derived parameters, several confidence checks are performed to ensure the software's accuracy and reliability.

**Table 1:** Baseline table, stratified by outcomes.

	Combined Outcome		
	No	Yes	
n	529	101	P value
Demographics			
Age	79.11 (7.20)	79.66 (8.06)	0.489
BMI	27.70 (5.42)	27.18 (5.77)	0.388
Sex (= Male)	254 (48.0)	49 (48.5)	1.000
Diabetes	151 (28.5)	35 (34.7)	0.265
Hypertension	297 (56.1)	60 (59.4)	0.619
COPD	116 (21.9)	32 (31.7)	**0.046**⁎
Atrial Fibrillation	163 (30.8)	46 (45.5)	**0.006**⁎
CAD	284 (53.7)	63 (62.4)	0.134
Previous MI	85 (16.1)	16 (15.8)	1.000
Previous PCI	192 (36.3)	39 (38.6)	0.741
CVA	77 (14.6)	13 (12.9)	0.773
CABG	95 (18.0)	22 (21.8)	0.444
PVD	71 (13.4)	20 (19.8)	0.129
Heart failure	159 (30.1)	44 (43.6)	**0.011**⁎
Pulm. HT	7 (1.3)	4 (4.0)	0.150
AV-Block	89 (16.8)	18 (17.8)	0.920
Pacemaker	47 (9.0)	3 (3.1)	0.079
(Active) Malignancy	44 (8.4)	14 (14.4)	0.095
Dialysis	51 (9.6)	10 (9.9)	1.000
Medications			
Betablockers	315 (59.7)	70 (69.3)	0.087
Calcium Antagonists	150 (28.4)	21 (20.8)	0.146
ACE/ARB	307 (58.1)	52 (51.5)	0.259
MRA	76 (14.4)	28 (27.7)	**0.002**⁎
Statins	318 (60.1)	56 (55.4)	0.444
Loop diuretics	223 (42.9)	59 (59.0)	**0.004**⁎
Other Diuretics	85 (16.8)	14 (14.0)	0.592
Insulin	64 (12.1)	14 (13.9)	0.748
Metformin	92 (17.5)	14 (13.9)	0.460
Anti-diabetics (other)	52 (9.9)	13 (12.9)	0.466
Aspirin	270 (51.1)	41 (40.6)	0.067
VKA	129 (24.4)	46 (45.5)	**<0.001**⁎
Immunosuppressive	41 (8.5)	10 (10.3)	0.716
NOAC	48 (9.1)	10 (9.9)	0.949
Clopidogrel (P2y12)	135 (25.6)	23 (22.8)	0.632
Biomarkers			
Sodium	140.00 [138.00, 141.00]	139.00 [137.00, 141.00]	**0.010**⁎
Potassium	4.30 [4.00, 4.60]	4.30 [4.00, 4.70]	0.707
Urea	7.60 [6.00, 9.70]	8.95 [6.80, 12.50]	**<0.001**⁎
Hb	7.96 (1.03)	7.72 (1.07)	**0.034**⁎
(Log-)NT-proBNP	6.99 (1.28)	7.73 (1.26)	**<0.001**⁎
Creatinine	90.00 [75.00, 110.00]	95.00 [80.00, 122.00]	**0.027**⁎
eGFR	60.96 (18.22)	57.72 (18.75)	0.106
Echocardiography			
IVSd, mm	11.19 (2.17)	10.83 (2.12)	0.180
LVPWd, mm	11.01 (2.18)	11.21 (2.31)	0.487
RWT, %	0.50 (0.13)	0.50 (0.14)	0.925
LV mass, g	182.71 (55.06)	192.16 (58.80)	0.217
MV E	93.39 (29.07)	92.72 (25.60)	0.871
MV A	100.11 (29.49)	92.04 (34.37)	0.111
e’ septal, cm/s	5.35 (1.83)	5.82 (2.13)	0.128


	Combined Outcome		
	No	Yes	
n	529	101	P value
e’ lateral, cm/s	7.17 (2.24)	7.53 (2.45)	0.368
TAPSE, mm	20.86 (4.52)	19.95 (4.36)	0.105
TR Vmax, cm/s	3.04 (0.81)	3.02 (0.72)	0.830
PASP, mmHg	45.00 (20.60)	45.45 (19.59)	0.878
s' septal, cm/s	5.20 (1.33)	5.35 (1.60)	0.525
s' lateral, cm/s	6.36 (1.81)	6.27 (1.76)	0.783
AVA, mm^2^	0.79 (0.32)	0.80 (0.27)	0.877
AoV Vmax, cm/s	3.96 (0.75)	3.67 (0.75)	**0.001**⁎
AoV Pmean, mmHg	38.30 (14.52)	32.75 (13.26)	**0.001**⁎
LVOT, mm	18.95 (2.39)	19.61 (2.36)	**0.033**⁎
LVEF (a2c) †	55.83 (13.10)	51.76 (15.90)	**0.025**⁎
LVEF (a4c) †	55.63 (13.79)	51.07 (13.72)	**0.009**⁎
LVEDV (a2c) †	89.85 (37.79)	97.92 (45.40)	0.122
LVEDV (a4c) †	103.53 (37.56)	106.58 (43.47)	0.531
LVESV (a2c) †	42.37 (28.93)	50.75 (37.50)	**0.040**⁎
LVESV (a4c) †	48.40 (29.49)	54.60 (33.08)	0.104
LAESV (a2c) †	71.07 (28.75)	76.17 (28.41)	0.184
LAESV (a4c) †	71.39 (24.90)	76.72 (28.64)	0.088
LV GLS (a2c) †	−14.82 (4.89)	−13.69 (5.86)	0.074
LV GLS (a3c) †	−15.61 (5.44)	−14.40 (5.14)	0.116
LV GLS (a4c) †	−15.04 (5.03)	−13.85 (5.11)	0.050
LA Reservoir (a2c) †	19.62 (11.68)	16.01 (11.56)	**0.027**⁎
LA Reservoir (a4c)†	18.11 (11.02)	15.28 (11.87)	**0.042**⁎

Values are shown as n (%) for categorical variables, and mean ± (SD) or median [IQR] for continuous variables.

ACE/ARB: Angiotensin-Converting Enzyme Inhibitor / Angiotensin Receptor Blocker, Anti-diabetics (other): Other medications for diabetes besides insulin and metformin, AV-Block: Atrioventricular Block, BMI: Body Mass Index, CAD: Coronary Artery Disease, CABG: Coronary Artery Bypass Graft, COPD: Chronic Obstructive Pulmonary Disease, CVA: Cerebrovascular Accident, eGFR: Estimated Glomerular Filtration Rate, Hb: Haemoglobin, (Log-)NT-proBNP: (Logarithmically transformed) N-terminal pro-B-type Natriuretic Peptide, Pulm. HT: Pulmonary Hypertension, MRA: Mineralocorticoid Receptor Antagonist, NOAC: Non-vitamin K antagonist direct oral anticoagulant, PCI: Percutaneous Coronary Intervention, MI: Myocardial Infarction, VKA: Vitamin-K Antagonist.

⁎ A *P* value of <0.05 was considered as statistically significant.

† LVEF, LVEDV, LV GLS and LA Reservoir strain are being averaged after imputation for prediction purposes.

### 2.5 Statistical analysis

Categorical variables were compared using either chi-square or Fisher's exact test and presented as n (%). Continuous variables were reported as mean ± (standard deviation (SD)) for normally distributed variables or median [Interquartile range (IQR)] in case of non-normally distributed variables, and *t*-tests or Mann-Whitney tests (if non-normally distributed) were used to perform statistical comparisons. (NTpro-) BNP levels were log-transformed before analysis to normalize the distribution. A two-sided *P*-value of <0.05 was considered statistically significant.

Variable selection was performed with LASSO (least absolute shrinkage and selection operator), which has been described before [20], [21] **(S. fig. 2)**. A total of 72 candidate variables were included, as listed together with missing data rates in **S.** Table 1**.** Missing data was multiple imputed when considered missing at random. EuroSCORE II was neither imputed nor considered during variable selection and components of left ventricular ejection fraction (LVEF), left ventricular/atrial end-systolic or end-diastolic volume (LAESV, LVESV, LVEDV), left ventricular global longitudinal strain (LV GLS) and left atrial (LA) Reservoir strain were being averaged after imputation for variable selection. After creation of 10 imputation datasets, variable selection was performed by LASSO Logistic regression on each imputed dataset, using the minimal lambda. A parameter was included in the final model when apparent in >5 imputed datasets, and the coefficients of the final model were pooled. (*n* = 1000) bootstraps performed internal validation, and performance was obtained by subtracting the optimism from the apparent performance for each imputed dataset [22].

Sensitivity analysis was performed to determine the accuracy of the model in different sub-groups, including AV Mean gradient (high (≥40 mmHg) versus low (<40 mmHg)), time-period (early (2009–2015) versus recent years (2016–2020)) and access type (transfemoral versus non-transfemoral approach). Accuracy for EuroSCORE I and II to predict for the combined outcome was performed. External validation was performed in the external Bern TAVI registry. Predictive performance of the model was determined by assessing the area under the ROC (receiver operating characteristics curve) (AUC). All statistical analyses were performed using R-Studio software (version 4.3.1).

## 3 Results

In total, 1001 patients underwent TAVI in the UMCG between 2009 and 2020. After exclusion of patients with an indication for TAVI other than aortic stenosis (*n* = 13), missing follow-up data (*n* = 83) and missing echocardiography data (*n* = 78), 827 patients were eligible for this study (mean age of 79.2 years (± 7.3) and 53% females) **(**Fig. 1**)**.

Symptomatic data at baseline and during follow-up was available in 782 patients. After one year follow-up, 101 (12% of total 827) patients had adverse events and poor symptom improvement, while 529 (64% of total 827) patients had symptom improvement without adverse events, making 630 patients eligible for the primary outcome analysis (Table 1, Table 2**)**. 71 (9% of total 827) patients did not improve symptomatically without adverse events, while 81 (10% of total 827) patients had adverse events but nevertheless achieved symptom improvement.

**Table 2:**
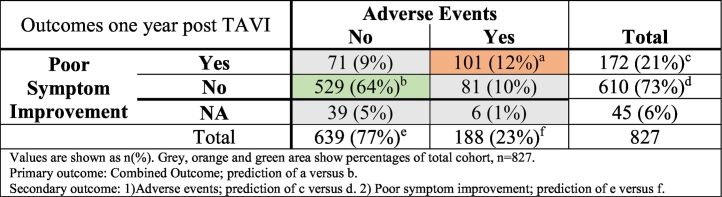
Outcomes one year post TAVI.

Values are shown as n(%). Grey, orange and green area show percentages of total cohort, *n* = 827.

Primary outcome: Combined Outcome; prediction of a versus b.

Secondary outcome: 1)Adverse events; prediction of c versus d. 2) Poor symptom improvement; prediction of e versus f.

### 3.1 Baseline characteristics

Patients with concomitant adverse events and poor symptom improvement were more likely to have a history of COPD, atrial fibrillation, and a history of heart failure, more often used mineralocorticoid receptor antagonist (MRA), loop-diuretics and vitamin-K antagonists (VKA) and had lower levels of sodium, higher levels of urea, lower haemoglobin levels, higher (Log-)NT-proBNP and creatinine levels, lower aortic valve (AV) Vmax, lower AV Mean Gradient, larger left ventricular outflow tract (LVOT) diameter, Lower LVEF (both apical two chamber (a2c) and apical four chamber (a4c) view), higher LVESV (a2c), and lower LA Reservoir Strain (both a2c and a4c view) at baseline.

### 3.2 Secondary outcome: poor symptom improvement or adverse events separately

In total, 172 (21% of total 827) patients did not improve in symptoms after TAVI **(**Table 2**).** 120 (14% of total 827) patients had died within one year. Adverse events were recorded in 188 (23% of total 827) patients, of whom 74 (11% of total 827) had died of cardiac cause, 102 (12% of total 827) were hospitalized for heart failure and 50 (6% of total 827) experienced stroke during 1 year follow-up **(S.** Table 2**).** Differences in outcomes between the two time-periods are shown in (**S.** Table 3).

**Table 3:**
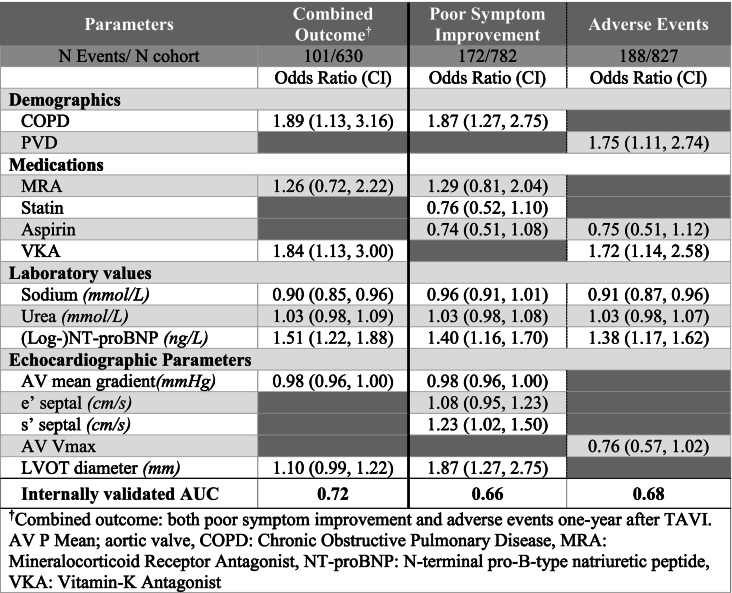
Prediction models.

†Combined outcome: both poor symptom improvement and adverse events one-year after TAVI.

AV P Mean; aortic valve, COPD: Chronic Obstructive Pulmonary Disease, MRA: Mineralocorticoid Receptor Antagonist, NT-proBNP: N-terminal pro-B-type natriuretic peptide, VKA: Vitamin-K Antagonist.

### 3.3 Predictors of the primary (composite) outcome

After LASSO Logistic regression variable selection predictors of the combined outcome were history of COPD (Odds Ratio (OR) 1.89, [95% Confidence Interval (CI): 1.13–3.16]), use of MRAs (1.26 [0.72, 2.22]), use of VKA (1.84 [1.13–3.00]) as well as lower sodium (0.90 [0.85–0.96]), higher urea (1.02 [0.98–1.09]), and higher (log-)NT-proBNP (1.50 [1.22–1.88]) levels and lower AV Mean Gradient (0.98 [0.96–1.00]) and higher LVOT Diameter (1.10 [0.99–1.22]). This model had an AUC of 0.74, which decreased to 0.72 after internal validation by bootstrapping **(**Table 3, graphical abstract).

### 3.4 Predictors for the secondary outcome

Predictors for the secondary outcome of poor symptom improvement were a history of COPD, use of MRA, use of statin, use of aspirin, use of VKA, higher levels of NT-proBNP and lower levels of AV mean gradient, but higher e’ septal and s' septal. Predictors for adverse events were history of PVD, use of aspirin, use of VKA, higher levels of NT-proBNP, lower levels of sodium and higher levels of urea together with a lower AV Vmax.

### 3.5 External validation and sensitivity analysis

In total, 3963 patients were included in the external validation cohort **(S. table 4).** Only the variables from the prediction model that were present in the validation cohort were included, resulting in a model consisting of history of COPD, use of VKA, AV-mean Gradient and (log-)BNP. For follow-up, heart failure hospitalisation was not routinely collected in that cohort. In the external cohort, the model achieved an AUC of 0.66 for predicting the primary outcome (**S. Table 5).** The model also showed similar AUCs for all different subgroups in the derivation cohort, including low/high gradient, time-period and TF versus non-TF. Applying the prediction model for the primary outcome to predict for the secondary outcome revealed slightly lower AUCs (**S. table 6).** The accuracy for the EuroSCORE to predict for the combined outcome was AUC: 0.64 (EuroSCORE I) and 0.59 (EuroSCORE II).

## 4 Discussion

The most important findings of this retrospective, observational cohort study with external validation were that in patients undergoing TAVI for a symptomatic aortic stenosis:

1) Most patients (64%) showed symptom improvement, without having adverse events, while 12% had both adverse events and poor symptom improvement after one year.
2) A model predicting for the combined outcome consisted of history of COPD, use of MRA, use of vitamin K antagonist, lower levels of sodium, higher levels of urea and (log-)NT-proBNP lower AV Mean gradient levels and larger LVOT diameter),
3) The model showed a validated AUC of 0.72 (Internal) with acceptable accuracy in an external, more recent cohort (0.66) and other subgroups.
4) The model is easy to use and can aid in clinical decision making and patient expectation management and potentially help avoid futile procedures.

A variety of TAVI outcome prediction models have been published in recent years, but the current model is novel and clinical applicable for the following reasons: first, it is easy-to-use, with only 8 (routinely measured) variables, making the model easy to interpret and collect. Secondly, it was built including 72 (clinical, pre-procedural) candidate variables with the addition of AI-echocardiographic parameters in the model to minimise the inter-observer variability. Thirdly, LASSO and Logistic Regression as methods for model variable selection have previously been shown to produce accurate prediction models [21]. While the accuracy of the model was good internally (AUC: 0.72) it showed modest accuracy in an external cohort (0.66). Lastly, the model showed the same modest accuracy in several sensitivity analysis, including high versus low gradient, different time-periods and access types. Lastly, it outperformed the EuroSCORE in predicting outcomes.

The incidence of symptom improvement in our study (73%) was lower compared to previous literature, which might be explained by our strict definition of symptom improvement [23]. The adverse outcome did occur in 23% of the patients, which is higher compared to major trials, but these trials might have a selection bias towards somewhat lower risk and healthier patients [24]. The adverse outcome was mainly driven by heart failure hospitalisations, which was defined as all heart failure hospitalisations, in contrast to only new or worsening heart failure in other studies. In addition, we also included patients who underwent a TAVI as long as 14 years ago, when event rate was higher than at present.

COPD as a predictor for the combined outcome is in line with previous literature and is commonly explained by the fact that patients with COPD have shortness of breath complaints not explained by the aortic stenosis, which did thus not improve after TAVI [23], [25]. This is supported by the finding that COPD was a predictor of poor symptom improvement but was not a predictor of adverse events separately.

Patients who used an MRA also had a greater likelihood of poor symptom improvement. While we do not have information regarding the exact indication of MRA, hypothetically, these patients most likely used an MRA since they were diagnosed with more advanced heart failure which did not recover after TAVI [26]. VKA use at baseline was a specific predictor of adverse events. This can be explained by the observation that VKA users are often patients with concomitant comorbidities such as atrial fibrillation, history of stroke or PVD [27]. In addition, MRA and VKA use can identify patients with the difficult to treat overlap syndrome of aortic stenosis with heart failure with a preserved ejection fraction (HFpEF) and atrial fibrillation [28], [29].

Lower sodium and higher plasma urea levels were predictors of the combined outcome, which was driven by their association with adverse events. The association between hyponatremia and mortality in TAVI patients confirms previous findings form the OCEAN-TAVR registry [30]. In heart failure patients, hyponatremia is a well-established marker for adverse clinical outcomes [31]. Higher urea levels per see have not been found as predictors for adverse events after TAVI, but may indicate renal impairment, which in turn has been identified as strong predictor of poor outcomes [32]. Higher urea levels are also associated with increased mortality in general aortic stenosis patients [33]. Higher NT-proBNP was both a predictor of adverse events and of poor symptom improvement. This might be explained by the notion that patients with heart failure (reflected by a higher NT-proBNP) both have a higher risk of adverse events and are less prone to recover after TAVI. [34].

A lower aortic valve (AV) mean gradient was associated with a higher risk of adverse events and poor symptom improvement. Patients with lower AV mean gradient most likely represent patients with a classical and paradoxical low-flow low-gradient (LF-LG) type of aortic stenosis. Both low flow types of AS have been associated with adverse outcomes as they may be caused by other comorbidities, resulting in higher mortality rates or the inability to improve symptomatically [35], [36]. The sensitivity analysis showed comparable accuracy of the prediction model for both low and high gradient patient populations.

The prediction model was externally validated in a large, contemporary patient cohort, with patients included until 2023. Although not all parameters were present in this cohort, the model still had modest predictive power. This may speak to the importance of the identified parameters, namely NT-proBNP levels and MRA use. These parameters should be collected in patients before TAVI to better inform them of benefits and risks of the procedure.

Symptom improvement is crucial in TAVI patients. If the likelihood of symptom improvement is low, physicians should reconsider the indication for TAVI. The risk of adverse events that a patient or physician is willing to accept may vary. Therefore, balancing these outcomes with a patient-specific approach is needed and predictors for these endpoints can help in clinical decision-making and help avoid potentially futile procedures. Patients at risk of adverse events and poor symptom improvement, can be identified as a high-risk category (12% of the patients in current analysis), that could potentially be considered for alternative therapies for aortic stenosis like Cardiawave, or concurrent (heart failure) medical therapy, including betablockers, angiotensin receptor–neprilysin inhibitor (ARNI), MRAs and SGLT-2 inhibitors [37], [38]. Our created risk prediction model can help in identifying high-risk patients and decision-making, and is available to use online: https://keesvanbergeijk.shinyapps.io/tavi_risk_calculator_final/

### 4.1 Limitations

This study, however, has some limitations. First, because of its retrospective observational nature, (symptomatic) outcomes were non-standardised and assessed by different clinicians. Due to unavailability, we did not include patient reported outcomes. In addition, the NYHA sometimes has a subjective and interobserver variability [39]. However, NYHA class is easy to assess and is widely used in clinical setting, and might therefore be a useful tool in nowadays clinical setting. Future research can include score like KCCQ or SF-12 in order to show its correspondence with clinician-reported outcomes. In addition, although we have included 72 potential predictors, there is a risk of residual confounding. For example, both microvascular dysfunction and cardiac amyloidosis have been associated with limited symptom improvement after TAVI, but were not assessed in the current analysis [40], [41]. To make the risk prediction model accessible for pre-procedural decision making, we did not include peri-procedural parameters, which have shown to have impact (predominantly short-term) outcomes. Inclusion of peri-procedural parameters in a pre-processing analysis, did not show to add accuracy to the prediction model and included the same parameters. External validation was performed with only a subset of the initial prediction model parameters and outcomes. However, accuracy was still modest, showing its potential use in external cohorts. Accuracy may increase when all parameters identified in the original cohort are included, and thus validation in other cohorts is necessary. Future prediction models could further increase accuracy by including larger patient populations.

## 5 Conclusion

A proportion of patients (12%) fail to achieve symptom improvement and have adverse events after TAVI. Our externally validated model combining baseline clinical variables and deep learning derived echocardiographic parameters can modestly predict for these outcomes. This prediction tool can play a role in clinical decision making and help avoid possibly futile TAVI procedures.

## CRediT authorship contribution statement

**Kees van Bergeijk:** Writing – review & editing, Writing – original draft, Visualization, Validation, Supervision, Software, Resources, Project administration, Methodology, Investigation, Funding acquisition, Formal analysis, Data curation, Conceptualization. **Constantijn (Stijn) Venema:** Writing – review & editing, Writing – original draft, Visualization, Validation, Supervision, Software, Resources, Project administration, Methodology, Investigation, Funding acquisition, Formal analysis, Data curation, Conceptualization. **Bob Ophuis:** Writing – review & editing, Writing – original draft. **Luca Plekkenpol:** Writing – review & editing, Writing – original draft. **Mara Tomei:** Writing – review & editing, Writing – original draft. **Hayman Al-Barwary:** Writing – review & editing, Writing – original draft. **Jasper Tromp:** Writing – review & editing, Writing – original draft, Project administration, Methodology, Investigation, Funding acquisition, Formal analysis. **Yoran Hummel:** Writing – review & editing, Writing – original draft, Visualization, Validation, Supervision, Software, Resources, Project administration, Methodology, Investigation, Funding acquisition, Formal analysis, Data curation, Conceptualization. **Wouter Ouwerkerk:** Writing – review & editing, Writing – original draft, Data curation, Conceptualization. **Ad van den Heuvel:** Writing – review & editing, Writing – original draft. **Hindrik van der Werf:** Writing – review & editing, Writing – original draft. **Yvonne Douglas:** Writing – review & editing, Writing – original draft, Conceptualization. **Dajiro Tomii:** Writing – review & editing, Validation. **Thomas Pilgrim:** Writing – review & editing, Validation. **Stephan Windecker:** Writing – review & editing, Validation, Conceptualization. **Adriaan Voors:** Writing – review & editing, Writing – original draft, Visualization, Validation, Supervision, Software, Resources, Project administration, Methodology, Investigation, Funding acquisition, Formal analysis, Data curation, Conceptualization. **Joanna Wykrzykowska:** Writing – review & editing, Writing – original draft, Visualization, Validation, Supervision, Software, Resources, Project administration, Methodology, Investigation, Funding acquisition, Formal analysis, Data curation, Conceptualization.

## Ethical statement

All studies have been approved by the appropriate ethics committee and have therefore been performed in accordance with the ethical standards laid down in the 1964 Declaration of Helsinki and its later amendments.

## Permission note

All material is original to this submission.

## Funding

Unrestricted research grant from 10.13039/100004374Medtronic and Us2.ai to UMCG.

## Declaration of competing interest

The authors declare the following financial interests/personal relationships which may be considered as potential competing interests:

Jasper Tromp is supported by the National University of Singapore Start-up grant, the tier 1 grant from the Ministry of Education and the CS-IRG New Investigator Grant from the National Medical Research Council; has received research support from AstraZeneca and consulting or speaker fees from Daiichi-Sankyo, Boehringer Ingelheim, Roche diagnostics and Us2.ai, and owns patent US-10702247-B2 unrelated to the present work. Yoran Hummel is employee of Us2.ai. D. Tomii has no conflict of interest. Thomas Pilgrim reports research, travel or educational grants to the institution without personal remuneration from Biotronik, Boston Scientific, Edwards Lifesciences, Medtronic and ATSens; speaker fees and consultancy fees to the institution from Biotronik, Boston Scientific, Edwards Lifesciences, Abbott, Medtronic, Biosensors, and Highlife. Stephan Windecker reports Research, travel or educational grants to the institution from Abbott, Abiomed, Amgen, Astra Zeneca, Bayer, Bbraun, Biotronik, Boehringer Ingelheim, Boston Scientific, Bristol Myers Squibb, Cardinal Health, CardioValve, Cleerly Inc., Cordis Medical, Corflow Therapeutics, CSL Behring, Daiichi Sankyo, Edwards Lifesciences, Farapulse Inc. Fumedica, GE Medical Systems, Gebro Pharma, Guerbet, Idorsia, Inari Medical, InfraRedx, Janssen-Cilag, Johnson & Johnson, Medalliance, Medicure, Medtronic, Merck Sharp & Dohm, Miracor Medical, Neucomed, Novartis, Novo Nordisk, Organon, OrPha Suisse, Pharming Tech, Pfizer, Philips AG, Polares, Regeneron, Sanofi-Aventis, Servier, Siemens Healthcare, Sinomed, SMT Sahajanand Medical Technologies, Terumo, Vifor, V-Wave, Zoll Medical. He is Advisory board member and/or member of the steering/executive group of trials funded by Abbott, Abiomed, Amgen, Astra Zeneca, Bayer, Boston Scientific, Biotronik, Bristol Myers Squibb, Edwards Lifesciences, MedAlliance, Medtronic, Novartis, Polares, Recardio, Sinomed, Terumo, and V-Wave with payments to the institution but no personal payments. The employer of AAV received consultancy fees and/or research support from Adrenomed, Anacardio, AstraZeneca, Bayer AG, BMS, Boehringer Ingelheim, Corteria, EliLilly, Merck, Moderna, Novartis, Novo Nordisk, Roche diagnostics, SalubrisBio.The employer of JJW received consultancy fees and/or research support from Medtronic, US2.ai, Boston Scientific, Abbott, Medis, Novo Nordisk, Sinomed, SMT and Meril.

The other authors report no disclosures.

## Appendix A Supplementary data

Supplementary material Image 1
