# Supplementary material for: Identification of patients at risk for adverse events and poor symptom improvement after transcatheter aortic valve implantation
Source: Am Heart J Plus. 2026 Feb 2;63:100735. doi: 10.1016/j.ahjo.2026.100735 (PMC12906061; doi:10.1016/j.ahjo.2026.100735)
Supplement: Supplementary file 1 — Supplementary material [file mmc1.docx]

Supplementary material

[**Supplementary Figure 1.** Definition of symptomatic response 2](#_Toc198304568)

[**Supplementary Figure 2.** Statistical Method 3](#_Toc198304569)

[**Supplementary Methods:** Definitions of medical history 4](#_Toc198304570)

[**Supplementary Table 1.** Included variables and Missing numbers 5](#_Toc198304571)

[**Supplementary Table 2.** Adverse events 6](#_Toc198304572)

**Supplementary Table 3.** Adverse events per time-period ....................................................7

[**Supplementary Table 4.** Baseline characteristics and outcomes Bern TAVI registry 8](#_Toc198304573)

[**Supplementary Table 5.** External **v**alidation and sensitivity analysis primary endpoint 9](#_Toc198304574)

[**Supplementary Table 6.** Sensitivity analysis of prediction model for primary endpoint predicting secondary endpoints 10](#_Toc198304575)

**Supplementary Figure 1.** Definition of symptomatic response

**
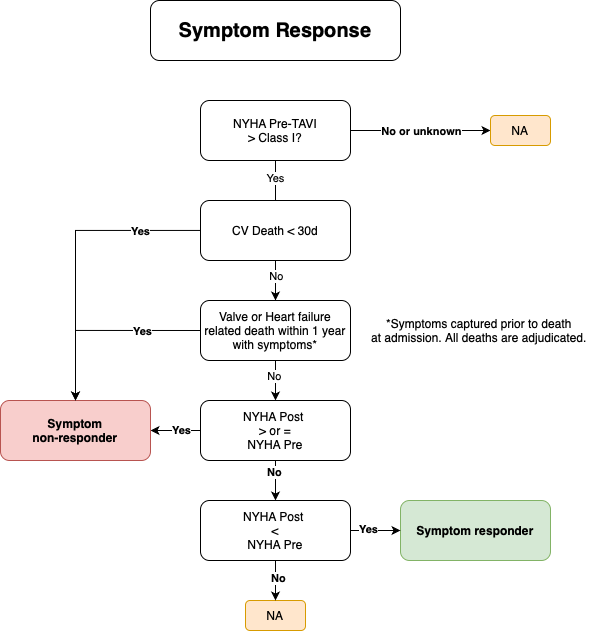
**

**Supplementary Figure 2.** Statistical Method

**
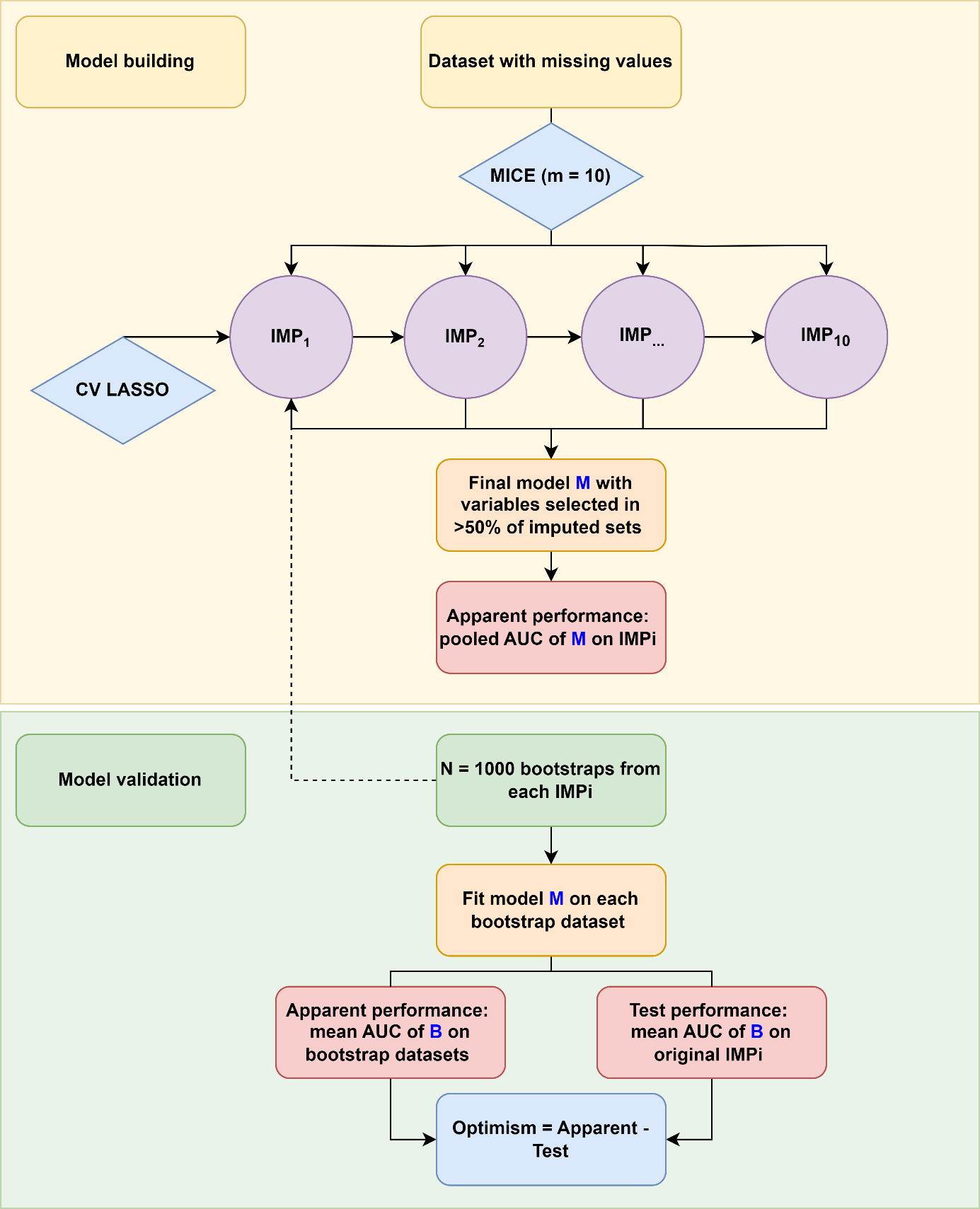
**

**Supplementary Figure 2.** **Statistical Method.** Flow chart summarizing the statistical method. The model was built after creating 10 imputed datasets. On each imputed dataset, LASSO penalized logistic regression was performed, and variables were included in the final model if apparent in >5 datasets. The apparent performance was measured by the AUC of the pooled model. Internal validation was performed by averaging the performance of (N=1000 times) bootstrapped samples of each imputed dataset and fitting the model on all bootstrapped sets.

AUC: Area under the curve, CV LASSO: cross-validating least absolute shrinkage and selection operator, MICE: Multivariate Imputation by Chained Equations, Imp_i_: imputation dataset (with number).

**Supplementary Methods:** Definitions of medical history

| Variable | Definition |
| --- | --- |
| Diabetes | Type 1 or 2 Diabetes |
| Hypertension | Documented in medical history |
| COPD | According to the Global Initiative for Chronic Obstructive Lung Disease (GOLD) report, includes all 4 GOLD stages |
| Atrial Fibrillation | All entities - paroxysmal, persistent and permanent - documented in the medical history or most recent ECG before the procedure. |
| CAD | ≥1 Significant lesion in all territories on most recent CAG. |
| Previous MI | Acute myocardial infarction, both STEMI and NSTEMI. |
| CVA | According to VARC-3 Criteria. |
| PVD | Defined according to local guidelines, based on an abnormal ankle-brachial index (ABI ≤0.9) |
| Heart failure | Any history of heart failure, documented in medical history and included both acute and chronic heart failure |
| Pulm. HT | Documented in medical history, according to VARC-3 Criteria. |
| AV-Block | Based on most recent ECG before the procedure. |
| Dialysis | Current dialysis. |

**Supplementary Table 1.** Included variables and Missing numbers

| Parameter | % Missing | Parameter | % Missing |
| --- | --- | --- | --- |
| Age | 0.0 | Potassium | 0.0 |
| BMI | 1.7 | Urea | 0.4 |
| Sex (= Male) | 0.0 | Hb | 0.0 |
| Diabetes | 0.0 | (Log-)NT-proBNP | 1.7 |
| EuroSCORE I | 51.1 | Creatinine | 0.1 |
| EuroSCORE II | 48.2 | eGFR | 0.6 |
| Hypertension | 0.0 | IVSd, mm | 27.8 |
| COPD | 0.0 | LVPWd, mm | 33.9 |
| Atrial Fibrillation | 0.0 | RWT, % | 37.5 |
| CAD | 0.0 | LV mass, g | 39.4 |
| Previous MI | 0.0 | MV E | 33.1 |
| Previous PCI | 0.0 | MV A | 44.0 |
| CVA | 0.0 | e’ septal, cm/s | 47.8 |
| CABG | 0.0 | e’ lateral, cm/s | 52.4 |
| PVD | 0.0 | TAPSE, mm | 23.7 |
| Heart failure | 0.0 | TR Vmax, cm/s | 33.0 |
| Pulm. HT | 0.0 | PASP, mmHg | 47.0 |
| AV-Block | 0.0 | s’ septal, cm/s | 48.6 |
| Pacemaker | 0.0 | s’ lateral, cm/s | 54.4 |
| (Active) Malignancy | 0.4 | AVA, mm^2^ | 47.6 |
| Dialysis | 0.0 | AoV Vmax, cm/s | 11.9 |
| Betablockers | 0.1 | AoV Pmean, mmHg | 11.9 |
| Calcium Antagonists | 0.1 | LVOT, mm | 36.9 |
| ACE/ARB | 0.1 | LVEF (a2c) | 34.6 |
| MRA | 0.5 | LVEF (a4c) | 25.5 |
| Statins | 0.0 | LVEDV (a2c) | 34.6 |
| Loop diuretics | 1.6 | LVEDV (a4c) | 25.5 |
| Other Diuretics | 4.2 | LVESV (a2c) | 34.6 |
| Insulin | 0.1 | LVESV (a4c) | 25.5 |
| Metformin | 0.2 | LAESV (a2c) | 36.2 |
| Anti-diabetics (other) | 0.2 | LAESV (a4c) | 25.0 |
| Aspirin | 0.1 | LV GLS (a2c) | 24.1 |
| Vitamin K antagonist | 0.1 | LV GLS (a3c) | 40.9 |
| Immunosuppressive | 9.1 | LV GLS (a4c) | 18.5 |
| NOAC | 0.2 | LA Reservoir (a2c) | 42.1 |
| Clopidogrel (P2y12) | 0.2 | LA Reservoir (a4c) | 29.7 |
| Sodium | 0.0 |  |  |
| Values are %  Total population N=827. | | | |

**Supplementary Table 2.** Adverse events

|  | CV Death | Stroke | HF Hosp | N |
| --- | --- | --- | --- | --- |
| N | 74 | 50 | 102 |  |
| Combination | No | No | Yes | 83 |
|  | No | Yes | No | 27 |
|  | No | Yes | Yes | 4 |
|  | Yes | No | No | 43 |
|  | Yes | No | Yes | 12 |
|  | Yes | Yes | No | 16 |
|  | Yes | Yes | Yes | 3 |
| % of total (n=827) | 8.9% | 6.0% | 12% |  |
| % of events (n=188) | 39.3% | 26.6% | 54.3% |  |
| CV Death: cardiovascular death, HF Hosp: heart failure hospitalisations | | | | |

**Supplementary Table 3.** Adverse events per time-period

|  | 2009–2015 | 2016–2020 | p |
| --- | --- | --- | --- |
| n | 326 | 501 |  |
| Combined Endpoint | 58 (17.8%) | 43 (8.6%) | **<0.001*** |
| Adverse Event | 102 (31.3%) | 86 (17.2%) | **<0.001*** |
| Stroke | 19 (5.8%) | 31 (6.2%) | 0.95 |
| CV Death | 45 (13.8%) | 29 (5.8%) | **<0.001*** |
| HF Hosp | 55 (16.9%) | 47 (9.4%) | **0.002*** |
| Poor Symptom Improvement | 75 (23.0%) | 97 (19.4%) | 0.24 |
| Incidence of events, stratified by year of procedure. Values are shown as n (%).  *A P value of <0.05 was considered as statistically signiﬁcant.  Values are n (%). CV Death: cardiovascular death, HF Hosp: heart failure hospitalisations | | | |

**Supplementary Table 4.** Baseline characteristics and outcomes Bern TAVI registry

|  | UMCG | Bern | p |
| --- | --- | --- | --- |
| n | 829 | 3963 |  |
| Age | 79.15 (7.30) | 81.79 (6.39) | **<0.001*** |
| Sex | 386 (46.7) | 2068 (52.2) | **0.004*** |
| NYHA (numerical) | 2.87 (0.60) | 2.67 (0.72) | **<0.001*** |
| NYHA Class |  |  | **<0.001*** |
| I | 27 (3.3) | 175 (4.4) |  |
| II | 131 (15.9) | 1353 (34.2) |  |
| III | 593 (71.8) | 2031 (51.3) |  |
| IV | 75 (9.1) | 401 (10.1) |  |
| COPD | 209 (25.3) | 430 (10.9) | **<0.001*** |
| Vitamin K antagonist | 232 (28.1) | 528 (13.3) | **<0.001*** |
| AV Mean Gradient *(mmHg)* | 37.17 (14.45) | 39.37 (16.78) | **0.001*** |
| (log-) BNP | *NA* | 5.76 (1.21) |  |
| (log-) NT-proBNP | 7.14 (1.29) | *NA* |  |
| Outcomes (1-year) | | | |
| Combined Outcome | 101 (16.0) | 330 (12.8) | **0.039*** |
| Poor Symptom Improvement | 172 (22.0) | 926 (28.0) | **0.001*** |
| NYHA Class (post-TAVI) | 1.51 (0.73) | 1.57 (0.70) | **0.022*** |
| Adverse Events | 188 (22.7) | 504 (12.7) | **<0.001*** |
| Mortality | 74 (8.9) | 309 (7.8) | 0.299 |
| Stroke | 50 (6.0) | 234 (5.9) | 0.940 |
| Heart failure hospitalisations | 102 (12.3) | *NA* |  |
| Values are shown as n (%) for categorical variables, and mean ± (SD) or median [IQR] for continuous variables.  *A P value of <0.05 was considered as statistically signiﬁcant.  AV: Aortic Valve, COPD: Chronic Obstructive Pulmonary Disease, (Log-)NT-proBNP: (Logarithmically transformed) N-terminal pro-B-type Natriuretic Peptide, NYHA: New York Heart Association | | | |

**Supplementary Table 5.** External **v**alidation and sensitivity analysis primary endpoint

| Grouping variable | Subgroup | AUC |
| --- | --- | --- |
| Complete Cohort | **Bern TAVI Registry** | **0.66** |
| AV Mean Gradient | Low (<40mmHg) | 0.73 |
|  | High (>40mmHg) | 0.75 |
| Time-period | 2009-2015 | 0.74 |
|  | 2016-2020 | 0.74 |
| Access-type | TF | 0.71 |
|  | Non-TF | 0.84 |
| EuroSCORE^†^ | EuroSCORE I | 0.64 |
|  | EuroSCORE II | 0.59 |
| ^†^EuroSCORE as predictor for the primary endpoint.  Numbers show accuracy by area under the curve (AUC) for predicting the primary endpoint with the prediction model of both absence of symptom improvement and adverse events. Subgroups of non-imputed datasets.  TF: transfemoral, Non-TF: direct-aortic, subclavian and transapical | | |

**Supplementary Table 6.** Sensitivity analysis of prediction model for primary endpoint predicting secondary endpoints

| Cohort | Adverse Event | Poor Symptom Improvement |
| --- | --- | --- |
| UMCG | 0.71 | 0.66 |
| Bern TAVI Registry | 0.60 | 0.62 |
| Numbers show accuracy by area under the curve (AUC) for predicting the secondary endpoints, by the prediction model created for the primary endpoint.  TF: transfemoral, Non-TF: direct-aortic, subclavian and transapical | | |
